# Lead-Free Perovskites for Lighting and Lasing Applications: A Minireview

**DOI:** 10.3390/ma12233845

**Published:** 2019-11-22

**Authors:** Elena V. Ushakova, Sergei A. Cherevkov, Vera A. Kuznetsova, Alexander V. Baranov

**Affiliations:** 1 Center of Information Optical Technologies, ITMO University, 49 Kronverksky pr., Saint Petersburg 197101, Russia; s.cherevkov@corp.ifmo.ru (S.A.C.); v.kuznetsova.a@gmail.com (V.A.K.); a_v_baranov@yahoo.com (A.V.B.); 2 Department of Materials Science and Engineering, and Center for Functional Photonics (CFP), City University of Hong Kong, 83 Tat Chee Avenue, Kowloon, Hong Kong, China

**Keywords:** perovskite, nanocrystals, lead-free, lasing, superluminescence, LED, photonics

## Abstract

Research on materials with perovskite crystal symmetry for photonics applications represent a rapidly growing area of the photonics development due to their unique optical and electrical properties. Among them are high charge carrier mobility, high photoluminescence quantum yield, and high extinction coefficients, which can be tuned through all visible range by a controllable change in chemical composition. To date, most of such materials contain lead atoms, which is one of the obstacles for their large-scale implementation. This disadvantage can be overcome via the substitution of lead with less toxic chemical elements, such as Sn, Bi, Yb, etc., and their mixtures. Herein, we summarized the scientific works from 2016 related to the lead-free perovskite materials with stress on the lasing and lighting applications. The synthetic approaches, chemical composition, and morphology of materials, together with the optimal device configurations depending on the material parameters are summarized with a focus on future challenges.

## 1. Introduction

The growing request of the market makes demands on modern photonics materials such as ability for miniaturization, higher efficiency, tunable and controllable optical and electrical properties, and stable performance. Thus, the scientific community tackles this challenge by the design and development of novel nanostructured optical materials. Among a wide variety of materials, perovskites can be highlighted thanks to their unique opto-electronic properties: High charge carrier mobility, high photoluminescence quantum yields (PLQYs), and high extinction coefficients, which can be tuned through all visible range.

Perovskite materials in common can be characterized by ABX_3_ chemical formula where A is a large monovalent organic or inorganic cation, B is a divalent metal cation, X is a halide anion, or their mixture. Although this formula is relatively simple, it allows one to form materials with different composition and, hence, to achieve a wide variety of physical and chemical properties. Other modifications of the perovskite crystal structure include:

- Layered or 2D, including Ruddlesden–Popper (RP) perovskites with formula L_2_BX_4_ where L is organic molecule (ligand),
- Double perovskites where the 2 B is replaced by 1 atom with 1+ valence and 1 atom with 3+ valence resulting in A_2_B^+^B^3+^X_6_ structure;
- Perovskite-related crystal structures with formula A_4_BX_6_ or AB_2_X_5_.

Starting from the first reports on the perovskite materials dated back to 1839 for the discovery of CaTiO_3_ mineral by Gustav Rose, the field was mostly devoted to the oxides with the same crystal symmetry until the investigation of cesium halides doped with Pb atoms which started in 1970s [1]. However, this topic was developed slowly and research was done only in the specific fields, since the PL quantum yield of the metal halide perovskites is quite low [2]. The first decade of XXIth century became a crucial moment of perovskite material history with observation of intense emission from the perovskite materials [3] and of extremely long lengths of charge carriers’ diffusion [4].

Considering the different morphology of perovskite nanomaterials, the two main classes can be highlighted: 2D or nanoplatelets and 0D or nanocrystals. The background for 2D perovskites was established in the 1990s and was related to the layered bulk crystals which possess similar properties as free-standing nanoplatelets. In 2015 Tisdale et al. [5] showed the synthesis of stable perovskite nanoplatelets, followed by work of Sichert and co-workers [6] on L_2_[MAPbBr_3_]_n−1_PbBr_4_ nanoplatelet synthesis with controlled thickness and optical transitions. Synthetic routes for nanocrystals were developed in parallel, and in 2014, a ligand-assisted reprecipitation method to synthesize the perovskite nanocrystals was first reported [7]; a hot-injection method of lead halide perovskite nanocrystal synthesis was first introduced by the work [8] describing a synthetic route of all-inorganic perovskite nanocrystals (NCs) with high PLQY. These works burst the interest of the scientific community and entailed the increasing number of published scientific and engineering works.

**Figure 1:**
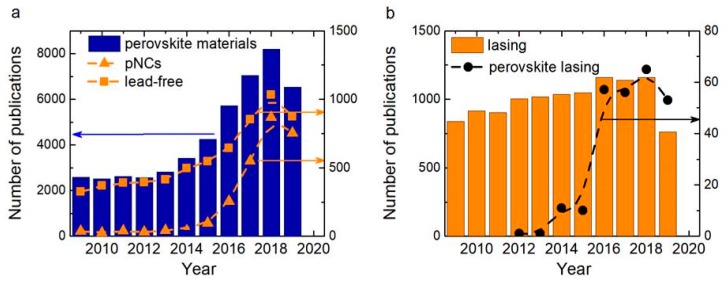
(**a**) Plot of publications number in the last decade: on perovskite materials (blue columns), on lead-free perovskite materials (orange squares), and on perovskite NCs (orange triangles). (**b**) Plot of publications number in the last decade: On lasing (orange columns), on lasing in perovskite materials (black circles).

Since 2009, more than 48,000 papers have been published already, and the amount of publication grows exponentially as it can be seen in Figure 1a. It is worth to mention that after 2015, a huge increase in the area devoted to perovskite nanocrystals was observed. To help a reader navigate in this ocean of information, several reviews are highlighted here to give a wide view on the perovskite-related topic.

In 2016, Q. Xiong et al. published a review [9] on synthesis and applications of metal halide perovskite nanomaterials. In this review, authors focused on precise and detailed description of the synthetic routes for nanocrystals, nanowires, and nanoplates formation, including hot-injection, ligand-assistant reprecipitation, and chemical vapor deposition methods with focus on the crystal formation mechanism. In 2018, M.V. Kovalenko, L. Manna, et al. in their review [10] highlighted the challenges and opportunities of colloidal lead-halide perovskite nanocrystals as a perspective materials to replace the conventional semiconductor quantum dots. Indeed, lead-halide perovskite NCs are believed to become as novel single-photon sources and highly efficient active down-converting media in lightning applications. The authors emphasized the existence of some ambiguities in the crystal structure, related energy structure, and contribution of different crystal phases and/or chemical composition to the NC emission. Along with the investigation of the perovskite nanocrystals, a lot of work was done in the research field devoted to 2D perovskite materials. In 2017, W. A. Tisdale et al. published a review on perovskite nanoplatelets [11], where they showed the 2D materials advantages over the bulk materials, and discussed in detail perovskite nanoplatelets synthesis, photophysics, and stability. Next, in 2018, Letian Dou et al. published a review [12] on 2D perovskite materials with extensive overview of their applications. It worth to be noted that yet another set of reviews were published in 2019 in Chemical Reviews [13,14,15,16], with the topics covered there including metal halide perovskite nanocrystals, layered halide perovskites, perovskite interfaces, and halide perovskite photovoltaics.

Although perovskite materials, in particular lead-halide perovskites, have already been implemented in different areas of photovoltaics, there are still some problems that need to be tackled. The main challenges are the increasing of the stability and mass production, and the development of the new lead-free materials. The publication analysis in Figure 1a shows that the number of papers on lead-free perovskites has also increased, especially over the last five years, and now reaches 14% from all number of papers. The solution of these problems will result in increased performance of the photovoltaic and optoelectronic devices based on the perovskite active media. In 2017, Zeijiao Shi et al. [17] reviewed the research field devoted to lead-free perovskite materials and revealed the main trends that need to be developed: Understanding of the fundamental photophysics and finding novel synthetic routes for lead-free perovskite materials. Since then, many scientific papers were published. We want to highlight in this piece some of the recent review papers on this topic.

In a recent review [18] by F. Sani et al., information on lead-free organic-inorganic halide perovskite solar cells was summarized. The authors inferred that the structural modification affects greatly the overall photovoltaic performance of the solar cells. Jia Sun et al. in their recent perspective [19] discussed different types of lead-free perovskite materials for future optoelectronic applications as LEDs and inferred that the working routes on the lead-based perovskites would benefit not only the developing field of light-emitting devices, but also the field of perovskite-based photovoltaics. Jiajun Luo et al. in their review [20] noted that although several types of lead-free perovskite materials were already established, there is still plenty of work to be done, including evolving the synthesis routes to achieve controllable PL peak position and PL QYs above 80%, improving the outdoor stability of operating devices, and expanding their applications towards luminescent solar concentrators and X-ray scintillators. In the lead-free perovskite family, 2D materials are highlighted by Jie Wang et al. in their recent review [21] with a focus on their perspective properties as highly luminescent phosphor for novel lightning applications.

Since the perovskite materials possess high emission quantum yields together with tunable optical transitions (properties) varying in a wide range, these materials are prospective in utilization as an amplifying media in laser systems. It is also confirmed by the increasing interest of scientific society shown by growth of publications, shown in Figure 1b. In a recent review [22] by K. Petridis et al., success in development of lasers based on organic and inorganic perovskite materials has been shown. In their review, operational principles and laser configurations were covered along with future challenges, including realization of electrically pumped perovskite lasers, stability of the optical responses during the device operating, and search for other chemical compound configurations of perovskite materials. M. L. De Giorgi and M. Anni in their review [23] focused on approaches to optimize the amplified spontaneous emission (ASE) properties in lead-halide perovskites including pumping regimes and configurations, including electrically pumped lasers.

In the present mini-review, we show recent development in the field of lead-free perovskite materials application in light-emitting devices. In the ‘synthesis’ section, we discuss in detail different synthetic approaches of fabrication lead-free perovskites with a focus on chemical composition and resulting morphology of materials. In the ‘lasing systems’ section, we discuss the morphology of perovskite materials utilized in the lasing devices with varied resonator’ architecture. In the last section, we discuss recent progress in LED and lasing technologies over the last five years. Finally, we give perspectives of the development of the lead-free perovskite materials for photovoltaic applications.

## 2. Synthesis

### 2.1. Chemical Composition

Since the perovskite materials are defect-tolerant, they possess high PL quantum yields of 60% for films [24], 26% for nanoplates [25], and up to 100% for NCs [8,26,27], which inspires scientists to design new lead-free materials with the similar crystal structure.

To replace Pb (II) in halide perovskites, several low-toxic cations from the same group of periodical table and those closest to it were proposed, including Sn(II) [28,29,30,31,32], In (III) [33,34], Bi(III) [35,36,37,38,39,40], Sb(III) [41], and others. At the same time, the double perovskite structure gained distribution in lead-free perovskites due to the wide range of cations from III group mixed with Ag(I) [42,43,44,45,46,47,48,49]. As for the lead-based, lead-free perovskites can also be divided to organic–inorganic and all-inorganic. For organic–inorganic lead-free perovskites, standard methylammonium [39,40,50], butylamine [32], 1,3-propanediammonium [51], and phenethylammonium [52] can be used as the A cations. For all-inorganic lead-free perovskites, the most used A cation is Cs, however there are several reports on Rb and Rb/Cs mixture [29,37]. The variety of chemical composition allows tuning the optical transitions of the lead-free perovskites in a wide spectral range from 425 to 990 nm. Here, we will discuss different chemical compositions of lead-free perovskite materials and describe the synthetic methods. Detailed information on the synthesis, morphology, and optical properties of lead-free perovskites is presented in Table 1. 

#### 2.1.1. Pb-Substituted Perovskites

• Tin

**Figure 2:**
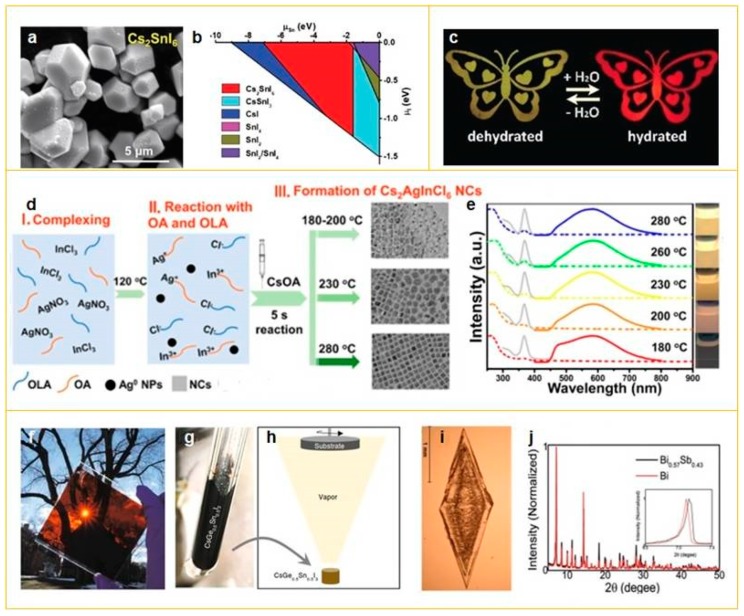
Synthesis of lead-free perovskites. (**a**) SEM images of Cs_2_SnI_6_; (**b**) calculated phase diagram of Cs–Sn–I. Reproduced with permission [31]. Copyright WILEY-VCH Verlag, GmbH & Co. KGaA, Weinheim, Germany, 2019. (**c**) Visualized dual emission between the hydrated and dehydrated species, fabricated by embedding the perovskite material, Cs_2_InBr_5_⋅H_2_O into an etched butterfly pattern. Reproduced with permission [34]. Copyright WILEY-VCH Verlag, GmbH & Co. KGaA, Weinheim, Germany, 2018. (**d**) Schematic representation on the phase formation of Cs_2_AgInCl_6_:Bi NCs at different synthesis temperatures (180–280 °C) in three steps: (I) Complexing of optimized precursors including OA, OlAm, and HCl; (II) AgNO_3_ and InCl_3_ reacting with OA to form Ag−oleate and In−oleate and oleylammonium chlorine. Ag^0^ NPs are formed by the reduction of Ag+ in the presence of amine ligands; (III) formation of Cs_2_AgInCl_6_ NCs at different synthesis temperatures followed by the injection of CsOA; (**e**) UV−Vis absorption (dashed line), photoluminescence (PL) (excitation at 368 nm), and PL excitation (emission at 580 nm) spectra of Bi-doped Cs_2_AgInCl_6_ NCs dispersion in hexane obtained at different synthesis temperatures (180–280 °C); the insets show the samples irradiated under a 365 nm UV lamp. Reproduced with permission [45]. Copyright American Chemical Society, 2019. (**f**) Photograph of an as-synthesized large-area CsSn_0.5_Ge_0.5_I_3_ perovskite thin film on a glass substrate showing dark reddish color. (**g**) Photograph of as-synthesized CsSn_0.5_Ge_0.5_I_3_ perovskite solid using the melt-crystallization method. (**h**) Schematic illustration of the single-source evaporation method for the deposition of ultrasmooth CsSn_0.5_Ge_0.5_I_3_ perovskite thin film. Reproduced with permission [56] Copyright Springer Nature Limited, 2019. (**i**) Image of (C_8_NH_12_)_4_Bi_0.57_Sb_0.43_Br_7_·H_2_O single crystal. (**j**) Powder X-ray diffraction (PXRD) patterns of (C_8_NH_12_)_4_Bi_0.57_Sb_0.43_Br_7_·H_2_O (black line) and (C_8_NH_12_)_4_BiBr_7_·H_2_O (red line). Reproduced with permission [52]. Copyright WILEY-VCH Verlag, GmbH & Co. KGaA, Weinheim, Germany, 2019.

Sn (II) attracted much attention due to its similarity of the electronic structure with lead atom. Thus, the tin-based perovskites are potentially applicable in the field of optoelectronic devices. In the work [31], a simple and efficient solution-phase method to synthesize Cs_2_SnX_6_ (X = Br, I) with a good yield, well-defined crystal structure, and long-term stability was demonstrated (Figure 2a,b). Cs_2_SnX_6_ single crystals show excellent stability against light and moisture due to the unique vacancy-ordered defect-variant structure, stable Sn^4+^ chemical compositions, as well as the lower formation enthalpy for Cs_2_SnX_6_. Another method [30] is hydrothermal synthesis of Cs_2_SnCl_6−x_Br_x_ millimeter-size single crystal, with the reaction mixture color continuously changing from transparent to yellow and, finally, to dark red. In addition, the band gap can be changed by the halide composition of single crystals, which leads to continuous tuning of the absorption spectra from near violet to orange spectral region. Also, for these structures, strong surface charge recombination of the excess carriers near the crystal surfaces created by short-wavelength light was observed.

The band gap of tin-based perovskite also can be altered by the changing the chemical composition of A cation. Mixed-cation perovskite system based on the substitution of cesium (Cs+) with rubidium (Rb+) in tin bromide perovskites Cs_1–x_Rb_x_SnBr_3_, has been experimentally demonstrated in [29]. The pure single-phase samples in compositional range of CsSnBr_3_–Cs_0.70_Rb_0.30_SnBr_3_ were obtained by high temperature (600 °C) synthesis and all the rubidium-embedding alloys showed a good stability. During chemical substitution from CsSnBr_3_ to Cs_0.70_R_0.30_SnBr_3_, the crystal lattice passes from cubic to orthorhombic symmetry, which was correlated with optical properties, since the band gap varies from 1.719 to 1.817 eV. To further boost the efficiency and stability of Sn-based perovskite, organic amine salts, such as butylamine (BA), were introduced into the crystal lattice [32]. This method allows more effective control of the crystallization kinetics of low-dimensional Sn perovskite films, due to which these structures acquire improved stability, homogeneity, and oriented crystal growth. Therefore, the crystallization kinetics jointly controlled by Lewis adducts and the ion exchange process using a mixture of ion liquid solvent methylammonium acetate and polar aprotic solvent dimethyl sulfoxide (DMSO) is demonstrated.

• Bismuth

Bi (III) is another non-toxic candidate element for a solution to the toxicity problem of lead-based perovskite materials. Methylammonium bismuth iodide ((MA)_3_Bi_2_I_9_) is one of most popular lead-free perovskite materials due to its air stability. Since the chemical composition of such a material is similar to organic–inorganic lead halide perovskites, the synthetic routes of film formation developed for lead-based perovskites, such as two-step deposition method, can be adopted for the Bi-based perovskites resulting in controllable morphology and high surface quality. Thus, in the work [39], the formation of (MA)_3_Bi_2_I_9_ films was demonstrated. Firstly, BiI_3_ was deposited onto the meso-TiO_2_ glass substrate followed by the toluene drop-casting and annealing, and secondly, the alcohol solution of MAI was spin coated and heated. For the hole transport layer, spiro-MeOTAD in chlorobenzene was deposited onto the prepared (MA)_3_Bi_2_I_9_ films. However, organic–inorganic perovskite films still contain the surface impurities, as pinholes. Wang’s group in their work [40] used diethyl ether as anti-solvent to improve the characteristics of (MA)_3_Bi_2_I_9_ based films such as compactness and reduce pinhole defects. The (MA)_3_Bi_2_I_9_ films treated by diethyl ether were compact with fewer pinholes and devices based on it exposited long-term air stability with 30% humidity for more than 200 days. The morphology of the perovskite film can also be improved by the post-synthetic treatment as was shown in work [36]. Stable (C_6_H_5_NH_3_)BiI_4_ perovskite film was formed by spin coating followed by the gas pump treatment, which resulted in a dense and pinhole-free film with lateral dimension more than 20 cm^2^. This material shows good solubility in ethanol and heat stability confirmed by thermogravimetric analysis. Also due to the presence of a hydrophobic organic chain, it was shown that the (C_6_H_5_NH_3_)BiI_4_ perovskite can withstand constant exposure of moisture for one year in ambient. Among the organic–inorganic bismuth-based perovskites, a (1,3-propanediammonium)_2_Bi_2_I_10_·2H_2_O with quantum-well morphology can be highlighted [51]. This material has sandwich structure with the inorganic Bi_2_I_10_^4−^ clusters periodically arranged in the crystallographic “c” axis separated by 0.5 nm layers of organic cations. The crystal growth is induced by the slow evaporation of precursors BiI_3_ and 1,3-propyl diammonium dihydrogen iodide.

The use of an inorganic cation at the A site of perovskite results in the increased stability and intensity of emission. In the work [38], a synthesis of double-halide single crystal Cs_3_BiBr_6_ perovskite was shown. The distinctive feature of double-halide perovskite materials is an isolated BiBr_6_ polyhedron in the crystal lattice. Therefore, the ratio between two raw precursors BiBr_3_ and CsBr plays key role in controlling the formation of the Cs_3_BiBr_6_ single crystals. It was also shown that such Cs_3_BiBr_6_ single crystals with band gap of 2.55 eV possess high thermal stability. To further improve PLQY of Cs_3_Bi_2_Br_9_ perovskite NCs, a Eu^3+^-doping can be used via modified ligand-assisted reprecipitation (LARP) method as was shown in work [53]. The Cs_3_Bi_2_Br_9_:Eu^3+^ NCs demonstrate multicolor emissions: Emission from the NC’ host Cs_3_Bi_2_Br_9_ matrix and the Eu^3+^ ion emissive transition. Compared to the Cs_3_Bi_2_Br_9_ NCs, the Eu^3+^-doped NCs show PLQY more than 40% and moisture stability. Alkali metals such as rubidium (Rb) have a good influence on boosting the optical characteristics of perovskites. Thus, in work [37], Rb_7_Bi_3_Cl_16_ single crystals with zero-dimensional cluster structure was reported. This structure represents two kinds of octahedra with different distortions. Such NCs were synthesized in a Teflon-lined stainless-steel autoclave, and they emit at 437 nm with a PLQY more than 28%. The moisture-stability of Rb_7_Bi_3_Cl_16_ NCs was attributed to increased ratio of Rb atoms and the [BiCl_6_]^3−^ octahedra on the surface, which formed an inorganic protective BiOCl shell.

• Indium

Another good candidate for lead atom substitution in the perovskite materials is indium. In the work [34], a hydrothermal synthesis of zero-dimensional indium-based Cs_2_InBr_5_⋅H_2_O single crystal with emission in red spectral region with PLQY of 33% was reported. The strong PL emission is due to self-trapping excitons, which are the result of structural deformation in an excited state. The transition between hydrated Cs_2_InBr_5_⋅H_2_O and the dehydrated form is accompanied by a switchable double emission, which allows detecting water molecules motion in air or organic solvents (Figure 2c).

• Other elements

Herein, we discuss some nontypical chemical elements that are used in lead-free perovskites. The Pb atom was substituted by Yb in a simple hot-injection synthesis of CsYbI_3_ NCs with high crystallinity and high uniform size distribution, reported in work [55]. Briefly, during the growth of nanocrystals, Yb (II) was introduced into the cubic ABX_3_ perovskite lattice. The synthetic route for formation of the CsYbI_3_ NCs is similar to that of lead halide perovskites, but with YbI_2_ used as a metal halide precursor. The synthesized NCs demonstrated strong excitation-independent emission and high PLQY of 58%. In this work [54], a synthesis of all-inorganic Cs_3_Cu_2_I_5_ perovskite crystalline film via a spin-coating method was reported. The Cs_3_Cu_2_I_5_ thin film was formed from CsI and CuI precursors sequentially added into a mixed solvent of dimethyl sulfoxide (DMSO) and dimethylformamide (DMF). The perovskite film exhibited pronounced sensitivity to deep UV and UV light illumination with response rise/fall time speeds of 26.2/49.9 ms. Another chemical element that can be used for substitution of lead atom is gold. Two new hybrid organic–inorganic gold perovskite-like halides, (CH_3_NH_3_)AuX_4_⋅H_2_O (X = Br and Cl) were reported in work [50]. Non-stoichiometric 2:1 molar ratio of methylammonium halides (MACl or MABr) and Au (III) halides (AuBr_3_ or AuCl_3_) in water/methanol 0.2 M solution system was used. The open flasks with mixture of precursors were kept at room temperature that allowed the evaporation of the solvent. After evaporation, dark red (CH_3_NH_3_)AuBr_4_⋅H_2_O crystals and yellow (CH_3_NH_3_)AuCl_4_⋅H_2_O crystals were obtained with lateral size up to 5 mm. These hydrated crystals formed in a new type structure featuring perovskite-derived 1D chains and 2D layers based on AuX_6_ pseudo-octahedral building blocks. At room temperature, both crystals show a weak blue emission, which originates from the electronic transition between Au-6s and Au-5d.

#### 2.1.2. Double Perovskites

The other big class of lead-free perovskites is the double perovskites where Pb (II) atom is substituted by pair of Ag (I) and Metal atom of 3+ valence. The most spread chemical composition for lead-free double perovskites is Cs_2_AgBiX_6_. In the work [44], the dependence of the energetic structure of Cs_2_AgBiBr_6_ thin films on the molar ratio of precursors CsBr:AgBr:BiBr_3_ was investigated. Precise solution processing allows obtaining accurate composition stoichiometry of Cs_2_AgBiBr_6_ multicomponent perovskite films, and hence good optoelectronic properties. The highly stable Cs_2_AgBiBr_6_ thin film was used as an active material for sensor due to the fact that its electrical properties are significantly dependent on humidity in [48]. Fast response and recovery of this Cs_2_AgBiBr_6_ thin film can be explained by the reversible physical adsorption of water molecules at the surface of thin film. A convenient solution method of synthesis to deposit high-quality Cs_2_AgBiBr_6_ film with long lifetimes, low trap densities, and large grain sizes was developed in work [49]. In this paper, authors optimized the precursor solution using a mixture of DMSO and DMF as the solvents and found that the introduction of a small amount of DMF is helpful to increase the grain size of the obtained Cs_2_AgBiBr_6_ films. As a result, the incorporated excess of DMF may change the ratio of precursors CsBr:AgBr:BiBr_3_ in the solution and prevent the AgBr to fit within the crystal lattice and to form the impurity in Cs_3_Bi_2_Br_9_. However, the use of 10% DMF resulted in formation of double perovskite film and the PL intensity increase with preserved absorption spectrum. This was accompanied with an increase in PL lifetimes, suggesting that reducing the trap densities is consistent with the enlarged grain size and improvement of crystallinity. It was recently shown [47] that these Cs_2_AgBiBr_6_ perovskite materials can be implemented in the photocatalytic system for dye degradation. It was found that during photocatalytic processes, Cs_2_AgBiBr_6_ is stable in ethanol. Acceleration of the reaction between free radicals and dye molecules indicates the unique catalytic properties of the Cs_2_AgBiBr_6_ surface. Also, the deposition of metal clusters, such as Pt onto Cs_2_AgBiBr_6_, effectively enhances the photocatalytic activity.

Along with Bi atoms, Sb and In halides are used in formation of lead-free double perovskites. In work [46], a double Cs_2_AgSbBr_6_ perovskite with an indirect optical bandgap of 1.64 eV was synthesized hydrothermally in aqueous HBr acid. This all-inorganic double Cs_2_AgSbBr_6_ perovskite crystallizes in the cubic space group Fm3m with a = 1.1 nm. After heat treatment at 200 °C, the crystallinity and symmetry were preserved. The atomic ratios of 2.18 (Cs):1.01 (Ag):0.91 (Sb):5.90 (Br) and existence of Sb (V) regions were found, which is consistent with the crystallographic composition and the charge transfer from the Sb (III) to Sb (V). In the work [45], the optimization of the colloidal hot-injection synthesis of undoped and Bi-doped Cs_2_AgInCl_6_ perovskite nanocrystals was described. The whole synthesis conditions such as temperature, number of ligands, and hydrochloric acid were evaluated to enhance the PLQY of Bi-doped Cs_2_AgInCl_6_ NCs (Figure 2d,e). The undoped nanocrystals demonstrated emission at 455 nm and the Bi-doped samples exhibit a broad emission peaked at 580 nm with the PLQY of ∼11.4%.

#### 2.1.3. Alloyed Perovskites

Another approach of substitution of Pb atoms in perovskite materials is a formation of alloyed perovskites with mixture of metal atoms at B site in crystal lattice. In the work [56], a synthesis of alloyed cesium tin-germanium triiodide (CsSn_0.5_Ge_0.5_I_3_) perovskite was shown (Figure 2f–h). The perovskite powder with this chemical composition was synthesized by a solid-phase reaction in evacuated Pyrex tubes between mixed CsI:SnI_2_:GeI_2_ solid powder precursors (molar ratio 2:1:1). It was also shown that due to the formation of a stable oxide layer, which fully encapsulated and passivated the perovskite surface, these powders and films exhibited very high stability. The surface-oxide passivation approach reported here represented an alternate way for increasing the stability and efficiency of lead-free perovskites. In the work [52], the synthesis of an air-stable zero-dimensional mixed metal halide (C_8_NH_12_)_4_Bi_0.57_Sb_0.43_Br_7_⋅H_2_O perovskite, in which [SbBr_6_]_3_ and [BiBr_6_]_3_ octahedral units are separated by the organic C_8_H_12_N^+^ cation, was reported. These single crystals (Figure 2i,j) exhibit moisture and light stability and show broadband emission ranging from visible to NIR region, which is caused by both free and self-trapped excitons.

### 2.2. Lead-Free Perovskites Morphology

The analysis of literature shows that lead-free perovskites can be formed as films [32,39,40,44,48,56,57], single crystals [25,26,29,32,33,41,42,44,45,46,47,50], and NCs [32,38,40,48,49]. The synthetic methods are similar to that used for lead-based perovskites. Spin-coating of the precursors on the substrates and annealing result in the formation of perovskite films with thickness up to 500 nm and grain size varying from 80 nm to 40 µm. Hydrothermal and induced crystallization methods, including antisolvent diffusion, vapor-assisted, cooling, and slow evaporation, result in the formation of single perovskite crystal with sizes varying from 250 nm to 1–2 cm. LARP and hot injection methods are used for NCs synthesis with diameter up to 10 nm and stabilized by long-chain ligands in non-polar solvents. It is worth mentioning that among the different morphologies of lead-free perovskite materials, the highest PLQY (up to 58% [55]) belongs to NCs, which is of importance for their utilization in lighting applications.

### 2.3. Stability

Although the lead-free perovskite materials have already found their implementation as an efficient photosensitive media for photodetectors and solar cells [18], these materials are still far from their commercial implementation due to their stability. This drawback becomes crucial when lead-free perovskites are applied as an emissive media in lasing systems and lighting applications. Since almost all chemical properties of lead-free perovskites are similar to that of Pb-based, the methods of stability increasing used for Pb-based can be adopted and implemented for lead-free perovskites. Several methods can be highlighted for this purpose: In-situ synthesis in polar environment [58], in-situ synthesis in protective matrices [59,60], the surface passivation by chemical post-treatment [61,62], and the embedding into the protective matrices [63]. The implementing of the host matrices is most appropriate for the further use in photonics application since this approach allows the in-situ design of the morphology of the active media, including size and shape, which is crucial for the performance of the emitting resonant devices, such as lasers.

Lead-free perovskites already implemented as a photosensitive material for photodetectors [64] and solar cells [18]. In addition, theoretical work [65] predicted that lead-free perovskites have even higher optical absorption compared to lead-based ones, which shows the advantages of their further implementation in all sorts of photovoltaic devices. In the following sections, we will focus on the light-emitting devices and lasers based on lead-free perovskites together with providing the background of the topic.

## 3. Lighting Applications

The principle of light emission by LED is based on the spontaneous recombination of electron–hole pairs in the active material, which is typically a semiconductor [66]. The radiative recombination can be carried through electroluminescence or photoluminescence. The latter is attributed to the down-conversion LEDs, where the emissive material or phosphor is excited optically by UV or blue chip. Taking into account such outstanding optical properties of perovskite materials as tunable in a wide range PL peak position, high PLQY, and small values of FWHM, there is no doubt that perovskites are promising for utilization as phosphors in LEDs. Indeed, just in the last few years, the luminance and external quantum efficiency of perovskite based LEDs increased their from 364 cd m^−2^ and 0.1% [3] to 76,940 cd m^−2^ and 16.5% [67], respectively. More information on recent developments of lead halide perovskites can be found elsewhere [68,69,70,71].

One of the first attempts to minimize the toxicity of lead-based perovskite materials was introducing the mixed-metal cation perovskites, thus decreasing the Pb atoms amount in the active material. In work [72] by Xiaoli Zhang et al., a set of CsPb_1−x_Sn_x_Br_3_ NCs were synthesized. It was shown that with increase of Sn content the absorption and emission bands are blue-shifted. The best performance of LED based on mixed-metal cation perovskite material was achieved for Sn content of 0.3 with maximum luminance of 5495 cd m^−2^.

**Figure 3:**
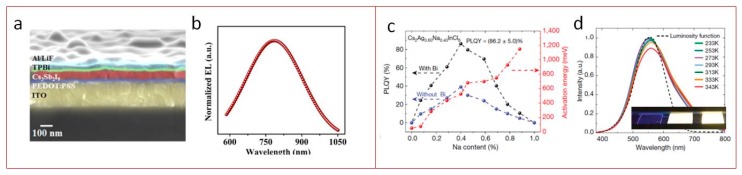
Lead-free film perovskite-based LEDs. (**a**) Cross-sectional SEM image of device; (**b**) normalized electroluminescence (EL) spectrum of the Cs_3_Sb_2_I_9_ film. Reproduced with permission [73]. Copyright American Chemical Society, 2019. (**c**) Activation energy and photoluminescence quantum yields (PLQY) of Cs_2_Ag_x_Na_1−x_InCl_6_ powder vs. Na content. The dashed lines are guides for the eye. (**d**) Cs_2_Ag_1−x_Na^x^InCl_6_ Luminosity function (dashed line) and photoluminescence spectra (solid lines) of Cs_2_Ag_0.60_Na_0.40_InCl_6_ measured at different temperatures from 233 K to 343 K. Reproduced with permission [74]. Copyright Springer Nature Limited, 2018.

Recently, an LED based on all-inorganic lead-free film emitter sandwiched between ITO/PEDOT:PSS and TPBi/LiF/Al as the hole and electron injection electrodes, respectively, was demonstrated in work [73] by Anupriya Singh et al. Authors showed that simple spin-coating method can be implemented to synthesize emissive Cs_3_Sb_2_X_9_ film, which PL can be tuned via vapor halide exchange method. A visible−infrared radiance of 0.012 W·Sr^−1^·m^−2^ was achieved at 6 V with Cs_3_Sb_2_I_9_ as active layer with electroluminescence peak at 750 nm (Figure 3a,b).

Jiajun Luo et al., at the end of 2018, published work [74] where authors showed that by the modification of the chemical composition of all-inorganic double perovskites, in particular, by alloying Na cations into Cs_2_AgInCl_6_ via hydrothermal method, resulted in suppression the dark transitions. Thus, highly luminescent powders were obtained with PLQY up to 86%. It was shown that optimally alloyed Cs_2_(Ag_0.60_Na_0.40_)InCl_6_ with 0.04% Bi doping perovskite emits warm-white light with luminance 5000 cd m^−2^ for over 1000 h (Figure 3c,d).

**Figure 4:**
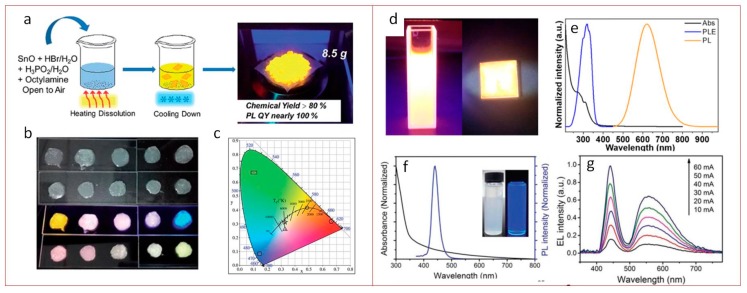
Lead-free perovskite nanomaterials based down-conversion LEDs. (**a**) Schematic representation of the synthesis of the 2D (OCTAm)_2_SnBr_4_ by a facile aqueous acid-based synthetic method in ambient air. (**b**) Images of yellow phosphors, blue/green phosphors, and their blends with different ratios embedded in PS films under sunlight (top panel) and 365 nm UV light (bottom panel). The first row in each panel are a yellow–blue phosphor mixture, and the second is a yellow–blue–green mixture. (**c**) Chromaticity coordinates of different ratios of the phosphor mixture plotted on the CIE1931 chromaticity chart: Blue phosphor (square), yellow phosphor (round), yellow–blue phosphor 4:1 (triangle), and yellow–blue–green phosphor 4:1:1.5 (star). Reproduced with permission [76]. Copyright Royal Society of Chemistry, 2019. (**d**) Photograph of the colloidal suspension and film of (OAm)_2_SnBr_4_ perovskites under UV light. (**e**) Normalized absorption (Abs), PL excitation (PLE, monitored at 620 nm), and PL (excited by 365 nm) spectra of the (OAm)_2_SnBr_4_ perovskite film. Reproduced with permission [75]. Copyright American Chemical Society, 2019. (**f**) Absorption and photoluminescence spectra of CsBr:Eu^2+^ NCs. The inset depicts the optical images of the NCs dispersed in hexane with and without illumination by a 365 nm UV lamp. (**g**) EL spectra and of the white LED operated under different forward bias currents. Reproduced with permission [77]. Copyright WILEY-VCH Verlag, GmbH & Co. KGaA, Weinheim, Germany, 2019.

Besides lead-free films and powders, nanomaterials with perovskite crystal structure also have found their lighting applications. Xiangtong Zhang et al., at the end of 2018, published a letter [75] on lead-free 2D Ruddlesden−Poppertype (C_18_H_35_NH_3_)_2_SnBr_4_ perovskite with high PLQY up to 88% in solution and 68% in the film. The 2D perovskites were synthesized by a hot injection method and passivated by oleylamine cations. Obtained films were further implemented as active media in the inverted orange LED showing a low turn-on voltage of 2.2 V and a luminance of 350 cd m^−2^ (Figure 4d,e). In 2019, Aifei Wang et al. [76] reported on synthesis of 2D (octylammonium)_2_SnX_4_ (X = Br, I) with >80% chemical yield produced by simple heating of the precursors in acid solution in ambient conditions. The synthesized material possesses 600 nm emission with near-unity PLQY. The position of the PL peak can be also tuned by halide exchange. This novel material was further used as a phosphor in down-conversion LED with white light emission (Figure 4a–c).

Zhiwen Yang et al. [77] developed a synthesis of bright blue emissive perovskite NCs inspired by hot injection method [8] with chemical formula CsBr:Eu^2+^ with an FWHM of 31 nm and PLQY of 32.8%. The authors showed principle opportunity to employ these NCs as a phosphor material for fabricating down-conversion white LEDs (Figure 4f,g).

## 4. Lasing

The term LASER is the abbreviation for “light amplification by stimulated emission of radiation”. Thus, lasers are light-emitting devices via a process of optical amplification based on the stimulated emission in the active (amplifying) medium. The first theoretical prediction of this phenomenon was conducted by Charles Townes and Arthur Schalow [78], which inspired further development of this research field. Since the first laser built by Theodor Maiman in 1960 [79,80], the number of lasers implementing different types of active medium together with the varied resonator’s geometries and pump sources significantly increased. These devices found applications in different fields from everyday life, for example CD/DVD to fabrication and scientific tasks such as microscopy with improved spatial resolution [81], etc.

### 4.1. Basic Principles

**Figure 5:**
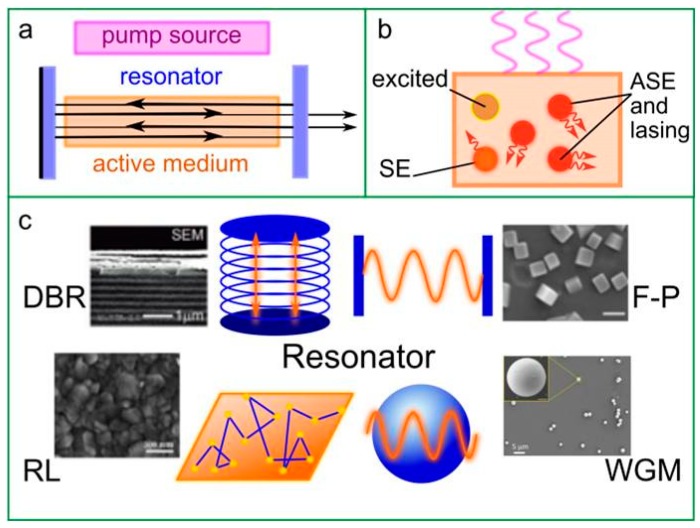
Architecture of perovskite-based lasers. (**a**) Simplified laser configuration. (**b**) Principle of stimulated emission occurred in active media: SE—spontaneous emission, ASE—amplified spontaneous emission. (**c**) Types of resonator’s geometries: DBR—distributed Bragg reflector, F-P—Fabry–Perot, RL—random lasing, WGM—whispery gallery mode. Insets in (c) are the examples of the morphology of perovskite materials used for DBR [82], F-P [83], RL [84], and WGM [85] resonators. Adopted with permission [82]. Copyright Royal Society of Chemistry, 2018; Adopted with permission [83]. Copyright American Chemical Society, 2018; Adopted with permission [84]. Copyright American Chemical Society, 2018; Adopted with permission [85]. Copyright Royal Society of Chemistry, 2019.

The basic and simplified laser configuration consists of the following elements (Figure 5a): (i) An active medium, where the stimulated emission process occurs; (ii) a pump source to produce the population inversion in active media; and (iii) a resonator, which is needed to stimulate the positive feedback mechanism that causes the majority of the atoms to contribute to the coherent output in active medium. The active medium should possess high gain coefficient, which makes possible stimulated emission amplification inside the material (Figure 5b). Briefly, the incoming photon from pump source excites the atom in active medium, and then it undergoes radiative relaxation through either spontaneous emission (SE) or stimulated emission of a coherent photon. The latter interacts with the incident photon from pump source which, in turn, causes the creation of doubled number of coherent photons. For the normal population of atoms, the SE prevails over the stimulated emission. To obtain the amplification, a population inversion is needed, which can be achieved by pumping the active medium. For that purpose, different sources are implemented so far. All variety of pump sources can be divided into two classes: Optical and electrical pumping. After excitation by pump, source atoms start to emit the photons in all directions and the photons that propagate along the active medium axis can stimulate other excited atoms to emit coherent photons. The “alignment” of emitted photons is achieved by different configurations of the resonators, for instance: (i) Distributed Bragg reflector (DBR), (ii) Fabry–Perot (F-P), (iii) random scattering (or random lasing, RL), and (iv) whispery gallery mode (WGM). The types of resonator’s geometry are depicted in Figure 5c.

The simplest resonator is F-P and is formed by two parallel mirrors, one of which is partially transparent (Figure 5a,c). This configuration allows the emitted light to propagate along the axis of amplifying materials, thus resulting in the amplification of the emission. In some cases, the initial reflectivity on the cleaved facets of semiconductor crystal served as active medium at the interface with air is sufficient enough to create a resonator. Most of the lasers based on F-P resonators are multimode. DBR–based resonator consists of multiple layers of materials with different refractive index (Figure 5c). On boundary between the layers, a constructive interference for waves with specific wavelength can occur. The efficiency of reflectance of a light emitted by an active medium in DBR resonator reaches 99%, thus this type of resonator is highly utilized in different laser systems such as vertical-cavity surface-emitting lasers (VCSELs) where quantum wells or thin films of luminescent material are used as active medium. WGM-based resonator is formed by a concave surface where a total optical internal reflection occurs for specific waves (Figure 5c). Thus, a light propagates inside the spherical cavity and amplifies by the interaction with the excited atoms in active medium. RL is not a resonator itself since the optical feedback in this case is provided by scattering particles in active medium, which can also emit light (Figure 5c). However, it is applicable for nanostructured materials with variable reflective index within material volume, for instance, for ensembles of QDs with high PLQY.

### 4.2. Perovskite Materials in Lasers

**Figure 6:**
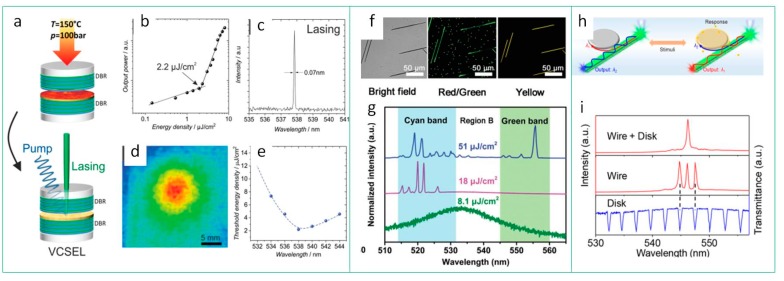
Resonators vs. perovskite material’s morphology. DBR: (**a**) Scheme of vertical-cavity surface-emitting lasers (VCSEL) device; (**b**) output power vs. pump energy density; (**c**) PL spectrum and (**d**) far-field beam distribution at pump energy density above threshold; (**e**) energy density of laser threshold energy vs. the PL peak position. Reproduced with permission [120]. Copyright WILEY-VCH Verlag, GmbH & Co. KGaA, Weinheim, Germany, 2019. F-P: (**f**) Microimages (bright field, green channel, yellow channel); (**g**) pump fluence dependent PL spectra of central region of CsPbBr_x_I_3−x_ nanowires. Reproduced with permission [125]. Copyright WILEY-VCH Verlag, GmbH & Co. KGaA, Weinheim, Germany, 2018. F-P combined with WGM disk: (**h**) Scheme of the switchable single-mode lasing from a perovskite microwire coupled with an organic microdisk; (**i**) transition from multimode to single-mode lasing of the typical MAPbBr_3_ nanowire coupled with a microdisk. Reproduced with permission [103]. Copyright American Chemical Society, 2018.

**Figure 7:**
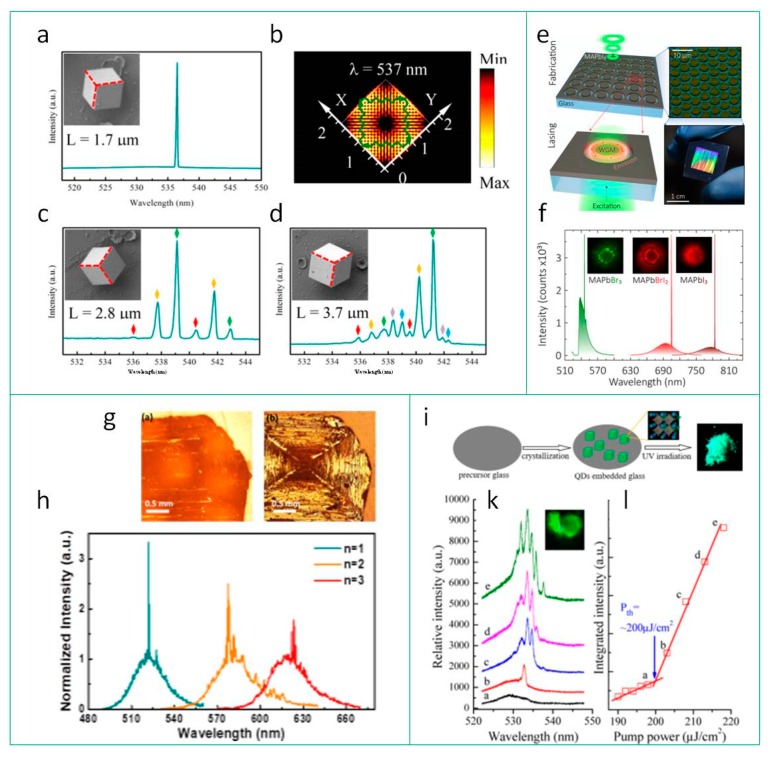
Resonators vs. perovskite material’s morphology. WGM: (**a**,**c**,**d**) lasing spectra of the CsPbBr_3_ microcuboids of different size, the insets are corresponding SEM images of the samples; (**b**) numerical simulations on the standing wave field distributions, which are confined in the square cross-section of cube with size of 1.7 µm. Reproduced with permission [96]. Copyright American Chemical Society, 2019. Array of WGM resonators: (**e**) Scheme of microlasers array fabrication, false-color SEM image, and a photo of a 1 × 1 cm^2^ array; (**f**) lasing spectra from perovskite microlasers from MAPbI_3_ (brown curve), MAPbBrI_2_ (red curve), and MAPbBr_3_ (green curve), the insets are PL images of the samples. Reproduced with permission [108]. Copyright American Chemical Society, 2019. RL: (**g**) Optical images of the (BA)_2_PbI_4_ (n = 1) single crystal bottom and top surface; (**h**) lasing spectra from high-quality homologous 2D perovskite single crystals with n = 1, 2, 3. Reproduced with permission [111]. Copyright American Chemical Society, 2018. (**i**) Scheme of a fabrication CsPbBr_3_ QD-embedded glass (QDs@glass) via in-situ crystallization; (**k**) up-conversion PL spectra of QDs@glass vs. the pump power of 800 nm laser, the inset is a photo of sample excited with energy density above threshold; (**l**) PL intensity vs. pump energy density. Reproduced with permission [118]. Copyright American Chemical Society, 2018.

To the best of our knowledge, the first observation of the lasing in perovskite materials is dated 1997 [86]. Takashi Kondo et al. reported on lasing of two-dimensional (C_6_H_13_NH_3_)_2_PbI_4_ perovskite thin film, which was achieved at 16 K and at 20 kW/cm^2^ laser pumping threshold. The main burst in this scientific area of utilizing the perovskite materials in lightning and lasing applications was caused by the synthesis of highly luminescent perovskite materials with QY above 80% [61,87]. At the present moment, the lasing perovskite materials possess wide range of morphology, including thin films [82,84,88,89,90,91,92], microstructures such as cubes [83,93,94,95,96,97], plates [81,98,99,100], wires [99,101,102,103,104], spheres [85,95,105], pyramids [97], nanosheets [106,107], microdisks [108,109], and quantum confined materials such as 2D R-P [110,111,112,113] and NCs [114,115,116,117], including NCs in glasses [118] and polymers [119]. Also, for the enhancement of the device performance, the perovskite materials can be patterned by ion beam lithography [109], laser ablation [108], or imprinting methods [89,120], and can be applied on the initially patterned substrates [81,90]. Perovskite-based lasers usually are optically pumped, which can be also up-conversion excitation of PL [83,84,92,96,106,116,118]. By the type of pump source operation, one can consider pulsed [57,82,92,109,112,113] and continuous wave (CW) [89,102,119,121] light sources used. The electrical type of pumping [122] is not so widely used, however, is still perspective. As in the case of conventional lasers, perovskite-based lasers are also formed with resonators such as DBR or waveguide [82,89,90,91,110,113,114,119,120] and F-P [94,97,101,102,103,104,114,123] (Figure 6), and WGM [57,85,93,95,96,99,100,105,106,107,108,109,115,124] and RL [84,88,92,111,112,116,117,118] (Figure 7).

From the analysis of recent publications, it can be inferred that the active material and corresponding type of resonator are connected to each other. DBR or VCSEL are suitable for the perovskite thin films, which can be easily deposited on the mirrors or waveguide surfaces via CVD or “wet” precursor spin-casting routes. In work [120], it was shown that the room temperature lasing can be achieved in CsPbBr_3_ thermally imprinted layers as active material at threshold down to 2.2 µJ cm^−2^ (Figure 6a–e). The single crystals, which are grown from the precursor solution on the substrates, can play role of the microcavity themselves. For instance, the perovskite microwires serve as the active medium and F-P resonator at the same time. In work [125], it was shown that composition-graded CsPbBr_x_I_3−x_ nanowires can be formed by vapor-phase growth on mica, which possess varied bandgaps along the wire and, hence, blueshifted emission observed at the wire’s ends (Figure 6f). In these wires, a double-color lasing is achieved with 35 nm separation between the emission bands, shown in Figure 6g. Since the F-P resonators provide a multi-mode generation, a controllable selection of lasing mode is in high demand for future tunable lasers. In work [103], the authors showed the implementation of organic microdisk with specific transmittance spectrum, which can be used as a filter for the lasing modes of perovskite microwires, as shown in Figure 6h,i.

By altering the shape of micro-sized perovskite materials, an emission enhancement by WGM can be achieved. Thus, WG modes are observed in microspheres, microcubes, microdisks, etc. (Figure 7a–f). In work [96], it was shown that by varying the length of the perovskite cuboid edge, a single- to multi-mode upconversion lasing is obtained at room temperature, which is also confirmed by numerical calculations of standing wave field distribution inside the perovskite cavity (Figure 7b). Thus, wavelength of lasing can be tuned from 534 to 544 nm via the size of the cavity (Figure 7a,c,d). In work [108], it was shown that the single mode lasing in the centimeter-sized arrays of perovskite microdisks can be achieved by the pattering the thin film via laser ablation (Figure 5e). Since the morphology of microdisks can be easily changed, it makes it possible to controllably tune the operating wavelength in the range from 550 to 800 nm (Figure 7f).

As for the conventional amplifying materials, the random lasing occurs in perovskite thin films with grain morphology, layered (R-P type) perovskites structure, or in ensembles of perovskite NCs, where the light is efficiently scattered on the structure boundaries. In work [111], it was shown that in 2D hybrid perovskite single crystals grown by slow evaporation method, a lasing is observed at room temperature (Figure 7g). The lasing wavelength can be tuned from 520 to 625 nm by engineering the perovskite layer thickness from one layer to three layers, respectively (Figure 7h). In work [118], it was shown that TeO_2_-based glass matrix with in situ synthesized CsPbBr_3_ NCs is a good candidate for implementing as an active medium in lightning devices (Figure 7i). Besides the photostability, moisture resistance, and thermal stability of such material, the RL were observed at room temperature pumped with relatively low threshold (Figure 7k,l).

The information from the publications starting from 2017 is summarized in Table 2 where the champion devices are shown. The analysis showed that the highest quality values, Q, are achieved for the single crystal perovskite microcavities with WGM resonator type. In [96], a Q = 10,100 was achieved in all-inorganic CsPbBr_3_ microcuboids with emission in green spectral region. The second efficient resonator in a frame of the highest Q value is VCSEL for which Q = 5400 was registered with CsPbX_3_ thin films used as active medium [120]. For lasers implementing F-P type resonators, the maximal Q value of 2500 was achieved for MAPbBr_3_ microcrystals [81]. For the RL case, the highest Q value of 1040 was registered for 2D R-P layers [111]. Thus, the synthetic routes, which determine the morphology of the nanostructured perovskite materials, also affect the type of a laser.

#### Lead-Free Perovskite for Lasing

**Figure 8:**
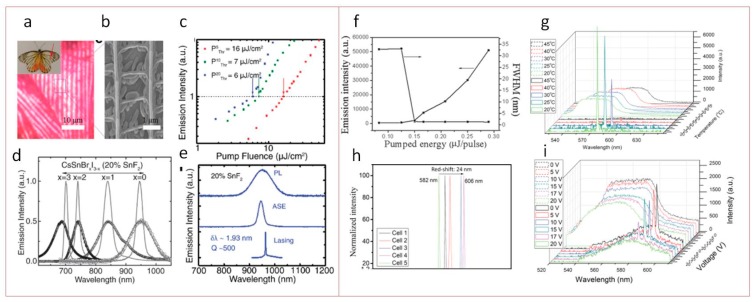
Lasing in lead-free perovskite materials. (**a**) Optical image of a butterfly scale from the white part of the wing. Inset is a photograph of the butterfly. (**b**) SEM image showing lamellae (vertical structures) in the scale. (**c**) Variable fluence measurements reveal the amplified spontaneous emission (ASE) thresholds of the SnF_2_-treated samples. (**d**) Wide PL and ASE wavelength tunability from CsSnBr_x_I_3−x_ films fabricated by facile mixing the precursor solutions under 500 nm pump pulses (50 fs, 1 kHz). (**e**) A comparison of the PL, ASE, and single-mode lasing of CsSnI_3_ (20% SnF_2_) under 650 nm pump pulses (50 fs, 1 kHz). Reproduced with permission [126]. Copyright WILEY-VCH Verlag, GmbH & Co. KGaA, Weinheim, Germany, 2016. (**f**) Variation in PL intensity and FWHM with pumped energy. (**g**) Thermal tunability of the AIPQD−CLC laser at E = 5.0 μJ/pulse at 20, 25, 30, 40, and 45 °C. (**h**) Tuning of the lasing emission of AIPQD−CLC laser by changing the chiral dopant content in the CLC. (**i**) Electrical tunability of the AIPQD−CLC laser at E = 4.0 μJ/pulse under AC voltages of 0−20 V (1 kHz). Reproduced with permission [127]. Copyright American Chemical Society, 2018.

Since the amplification of the stimulated emission imposes high demands on the active medium, lead-free perovskites are still not widely implemented in lasing systems. In 2016, Guichuan Xing et al. published their work [126] on NIR lasing from tin-based perovskite. They showed that tin-based halide perovskites (CsSnX_3_, X = Br, I) surprisingly possess exceptional optical gain properties in the near-infrared region up to ≈1 μm, which cannot be achieved with lead-based perovskites. NIR lasing with Q ≈ 500 is achieved using the natural photonic crystal at ultralow-threshold (≈6 μJ cm^−2^) from 20% SnF_2_-added to CsSnI_3_ samples, which are comparable to their lead-based counterparts (Figure 8a–e). This work confirmed the possibility of lead-free perovskites implementation in the lightning and lasing application despite poor photovoltaic performance.

In the work [127] by Lin-Jer Chen, a performance of laser based on cholesteric liquid crystal (CLC) doped with CsSnI_3_ QDs (AIPQD−CLC laser) with highly spectral tunability and long-term stability was demonstrated. The CsSnI_3_ QDs were obtained through a low-cost solvothermal pretreatment process with PL peak at 594 nm with FWHM of 35 nm. Under the optical pump with energy of 0.15 μJ/pulse, FWHM of PL peak decreased down to 0.20 nm with significant PL intensity increase (Figure 8f). These parameters are comparable to those of traditional dye-doped CLC lasers. It was shown that the laser based on lead-free perovskite QDs possessed high stability during storage in ambient (room temperature and humidity of 60%) and retained ~87% of its initial lasing efficiency after half a year. One of the interesting findings is that the lasing wavelength can be tuned by various approaches: Modification of the ratio of the chiral dopant in liquid crystal, changing the temperature, or applying different alternating current (AC) voltages to the active media in the laser (Figure 8g–i).

## 5. Outline and Perspectives

In the present mini-review, we briefly analyzed the state of art in the field of R&D of organic-inorganic and all-inorganic lead-free perovskites as crystal materials of different dimensionality with improved functional (detection and emission) properties for modern nanophotonics and photovoltaics. The main attention was paid to the recent publications on the development of active media based on lead-free perovskite materials for light sources demonstrating stimulated emission (lasing). The choice and design of laser resonators, influence of morphology of the perovskite active media, and methods for tuning the wavelength of laser emission were discussed. The almost 100% emission QY, possibility to change the lasing wavelength by changing the perovskite chemical composition and morphology, as well as by choice of type and design of resonators make perovskite-based active media very attractive for implementing in laser and lighting sources. It is important to mention that synthetic routes and device fabrication previously developed for lead-based perovskite materials can be efficiently inherent and adopted for their lead-free relatives, hence paving the way to perovskite “green” photonics. Although the number of scientific papers devoted to lead-free perovskite materials have increased in the past few years, there is still plenty of room for further development and improvement of their optical and electronic properties. According to the literature, at least 600 lead-free compounds with double perovskite crystal structure have not been investigated yet [16]. Along with the variety of chemical composition of lead-free perovskite materials, the chemical approaches of their synthesis and passivation are still unexplored. Thus, we highlight perspectives in the field of lead-free perovskites for lighting applications:

- Chemical composition and morphology. To date, Bi-based perovskite materials provide a wide variety of morphology of materials with perovskite symmetry, including double perovskites, along with the excellent and stable performance. The other direction is the search of novel chemical compounds for substituting the lead atom in lanthanide series, such as Yb;
- Synthetic approaches. Inspired by the chemical routes implemented for lead-based perovskites, the vapor deposition approaches might be admitted as synthesis route for formation micrometer-sized lead-free perovskites, the shape of which can meet the resonance conditions for stimulated amplification of the emission;
- Active medium protection. The use of the matrices for in-situ synthesis or as host matrices for perovskite materials can expand the variety of chemical compounds used, i.e., unstable in ambient, together with simple control of the architecture of the active medium defined by matrix morphology.

Thus, lead-free perovskite materials as elements of “green” technology are expected to be intensively studied and further applied in the light emitting devices in nearest future.

## Figures and Tables

**Table 1:** Chemical formula, morphology, and optical properties of lead-free perovskite materials and their synthesis routes.

Chemical Formula	Morphology	Size *	Type of Synthesis	Abs, nm	PL, nm	PL FWHM, nm	PLQY, %	Reference
**Tin-Based Perovskites**								
Cs_1–x_Rb_x_SnBr_3_	Film	N/A	Annealing	670–750	680–710	50	N/A	[29]
Cs_2_SnCl_6−x_Br_x_	Single crystal	1–2 cm	Hydrothermal	350–750			N/A	[30]
Cs_2_SnX_6_ (X = Br, I)	Single crystal	3–5 µm	Hydrothermal	900	675	50	N/A	[31]
BA_2_MA_3_Sn_4_I_13_	Film	GS 9 µm	Spin-coating	850	990	75	N/A	[32]
**Bismuth-Based Perovskites**								
(C_6_H_5_NH_3_)BiI_4_	Film	area > 20 cm^2^	Spin-coating	525	690	30	N/A	[36]
Rb_7_Bi_3_Cl_16_	Single crystal	18.4 × 7.2 × 6.0 mm	Hydrothermal	280	437	93	28.4	[37]
Rb_7_Bi_3_Cl_16_	NCs	1.85 nm	LARP	280	437	93	28.4	[37]
Cs_3_BiBr_6_	Single crystal	30 µm	Annealing	485	475	52	19.4	[38]
(CH_3_NH_3_)_3_Bi_2_I_9_	Film	GS 7.57 μm	Spin-coating	650			N/A	[39]
(CH_3_NH_3_)_3_Bi_2_I_9_	Film	GS 200 nm	Spin-coating	600	575 and 605		N/A	[40]
(PrAm)_2_Bi_2_I_10_·2H_2_O	Single crystal	1–10 mm	Antisolvent diffusion	600	530 and 690		N/A	[51]
Cs_3_Bi_2_Br_9_	NCs	3.3 nm	Hot injection	390	600 and 620		42.4	[53]
**Other Elements (In, Au, Cu, Yb)**								
Cs_2_InBr_5_⋅H_2_O	Single crystal	2 mm	Hydrothermal	450	695	200	33	[34]
(CH_3_NH_3_)AuX_4_⋅H_2_O, X = Cl, Br	Single crystal	5 mm	Slow evaporation	650	425	75	N/A	[50]
Cs_3_Cu_2_I_5_	Film	GS 40 µm	Vapor-assisted	400	442	100	N/A	[54]
CsYbI_3_	NCs	9.5 nm	Hot injection	660	671	50	58	[55]
**Double Perovskites**								
Cs_2_AgInCl_6_	NCs	10 nm	Hot injection	300	550	250	N/A	[43]
Cs_2_AgSbCl_6_	NCs	10 nm	Hot injection	360	550	250	N/A	[43]
Cs_2_AgBiBr_6_	Film	T 200 nm	Spin-coating	440	620	50	N/A	[44]
Cs_2_AgInCl_6_	NCs	10 nm	Hot injection	390	580	125	11.4	[45]
Cs_2_AgSbBr_6_	Single crystal	1 mm	Hydrothermal	550			N/A	[46]
Cs_2_AgBiBr_6_	Single crystal	1–10 µm	Hydrothermal	650			N/A	[47]
Cs_2_AgBiBr_6_	Film	GS 500 nm	Spin-coating				N/A	[48]
Cs_2_AgBiBr_6_	Single crystal	250 nm	Hydrothermal	440	630	145	N/A	[49]
**Alloyed Perovskites**								
CsSn_0.5_Ge_0.5_I_3_	Film	T 200 nm, GS 80 nm	Pyrex tubes annealing	840	830	52	N/A	[56]
(C_8_NH_12_)_4_Bi_0.57_Sb_0.43_Br_7_⋅H_2_O	Single crystals	3 mm	Cooling-induced	850	450 and 640		N/A	[52]

* T—film thickness, GS—grain size.

**Table 2:** Lasing parameters (type of resonator, pump source, lasing threshold, Q) vs. type of perovskite material (chemical formula, morphology, emission parameters) for the samples with the best performance.

Chemical Formula	Type of Perovskite	Type of Resonator	Pump Source	Lasing Threshold (CW—kW/cm^2^; P—µJ/cm^2^)	Spontaneous Emission		Stimulated Emission		Q max	Ref.
					PL Peak, nm; PLQY	FWHM, nm	PL Peak, nm	FWHM, nm		
(C_6_H_13_NH_3_)_2_PbI_4_	thin film	DBR	337 nm, 3 ns	20 @ 16 K	543	N/A	544	2	272	[86]
MAPbI_3_	nanoimprinted thin film	DFB	CW 355 nm	13·10^−3^	780	N/A	780	1.16	672	[89]
MAPbI_3_	thin film on the grating	DFB	532 nm, 1 ns	235	780	50	784	0.4	1960	[90]
MAPbX_3_	NCs	DFB	CW 405 nm	15–58·10^−3^	515/540; 95–97%	30	538.7	0.45	1200	[119]
CsPbX_3_	thin films	VCSEL	355 nm, 0.3 ns, 1 kHz	2.2	538; 68%	20	538.3	0.07	5400	[120]
MAPbBr_3_	microcrystals	F-P	400 nm, 100 fs, 1 kHz	9.1	530	40	548	0.21	2500	[81]
CsPbBr_3_	nanocuboid	F-P	400/800 nm, 35 fs, 1k Hz	40.2	530	18	539.2	0.29	2075/1859	[83]
CsPbBr_3_	nanowires	F-P	CW 450 nm	6 @ 77 K	530	15	533	0.25	2300	[102]
(OA)_2_(MA)_n−1_Pb_n_Br_3n+1_	2D R–P layers	F-P	400 nm, fs	8	530; 65%	30	545	0.3–0.6	1815	[123]
CsPbBr_3_	microcuboids	WGM	400 nm, 40 fs, 10 kHz	16.9	525	15.6	540.9	0.064	8500	[95]
CsPbBr_3_	microcuboids	WGM	800 nm, 40 fs, 10 kHz	210	528	20	536.8	0.053	10,100	[96]
CsPbBr_3_	microspheres	WGM	800 nm, 120 fs, 76 MHz	3.5 @ 300 K 0.4 @ 77 K	530	20	535–540	0.15	3600	[85]
(C_4_H_9_NH_3_)_2_(CH_3_NH_3_)_n−1_ Pb_n_I_3n+1_ (n = 1, 2, and 3)	2D R–P layers	RL	374 nm, 55 ps, 40 MHz	2.85	520–630	20	520–630	<0.5	1040	[111]
(BA)_2_(MA)_n−1_Pb_n_I_3n+1_	2D layers	RL	400 nm, 80 fs, 1 kHz	2.3 @ 70 K	520–680	15	520–680	0.9	755	[112]
MAPbBr_3_	thin films	RL	800/400 nm, 35 fs, 1 kHz	0.15 @ 300 K	535/545	30	550	5	110	[92]
CsSnX_3_ (X = Br, I) doped with SnF_2_	Film	DFB	650 nm, 50 fs, 1 kHz	6	680–950; 13%	50–100	950	1.93	500	[126]
CsSnI_3_	NCs in CLCC	DFB	532 nm, 8 ns, 10 Hz	800	594	35	579–606	0.2	2895	[127]
